# 

**Published:** 1892-10

**Authors:** 


### Books and Pamphlets Received.

Eleventh annual report of the State Board of Health of Illinois, Springfield, 1892.

Leopoldina, Arntleches organ der Kst Leop-Carol. Deutschen Akademie der Naturforscher. Halle, 1891.

Zur Kenntuess der Fettfar b'sloff—production bei Spaltpilzen von Dr. A. Overbeck. Halle, 1891.

Il Naturalesta Siciliano. Palermo, 1892.

Report on the Technical Determination of Zinc. Read before the Colorado Scientific Society, 1892.

Ottawa Naturalist.

Sitzungsbericht des Aerztlichen Vereins. Munchen, 1892.

Bulletin de la Societe Vandoise des Sciences Naturelles. Lausarme, 1892.

Archives Neerlandaises des Sciences Exactes et Naturelles. Harlem, 1892.

Bulletin de la Societe Linneenne der Nord de la France. Amiens, 1892.

Journal of Cincinnati Society of Natural History.

Bollettino dei Musei di Zoologia ed Anatomia comparata della R. Universita di Torino. Turin, 1892.

